# Divergent PICU lengths of stay despite identical phoenix sepsis scores: heterogeneity in pediatric sepsis outcomes according to system involvement profiles

**DOI:** 10.3389/fped.2026.1986840

**Published:** 2026-10-21

**Authors:** Zhen Jiang, Lijuan Wu, Xinyue Zhou, Jing Xu, Xiaxia Xiong, Jian Chen, Yuhang Hu

**Affiliations:** Department of Pediatric Critical Care Medicine, Sichuan Provincial Maternity and Child Health Care Hospital, Chengdu, Sichuan, China

**Keywords:** invasive mechanical ventilation, length of stay, pediatric sepsis, phoenix sepsis score, respiratory failure

## Abstract

**Objective:**

This study examined how different subsystem involvement patterns in the Phoenix Sepsis Score relate to PICU length of stay among children with sepsis, focusing on patients with identical total scores, and explored clinical factors associated with these differences.

**Methods:**

We retrospectively included 107 children with sepsis admitted to a single PICU (2024–2025). Patients were grouped by the number of affected Phoenix subsystems (respiratory, coagulation, circulatory, neurological): single-system (*n* = 37), dual-system (*n* = 62), and multi-system (*n* = 8). We further compared the largest subgroups with identical total scores of 2—isolated respiratory involvement (*n* = 29) versus respiratory-coagulation involvement (*n* = 59). Multivariable logistic regression identified predictors of prolonged PICU stay (≥10 days), and the association between IMV and PICU length of stay was further explored.

**Results:**

Respiratory and/or coagulation involvement accounted for 89.7% (96/107) of cases, with the respiratory–coagulation combination being most frequent (55.1%). The multi-system group had the highest total scores, lactate levels, and mortality (37.5%, all *P* < 0.05). Despite identical total scores [2 (2, 2), *P* = 0.457], the isolated respiratory group had significantly prolonged PICU stay [10 (9, 12) vs. 6 (4, 8) days, *P* < 0.001], higher ventilation rates (100% vs. 25.4%, *P* < 0.001), and longer ventilation duration [6 (4, 7) vs. 0 (0, 0) days, *P* < 0.001]. Invasive ventilation was the strongest risk factor for prolonged stay (OR=18.80, 95% CI: 3.78–123.24, *P* < 0.001). Adjusting for ventilation attenuated the group effect from OR=6.92 (*P* < 0.001) to OR=1.27 (*P* = 0.739), suggesting that the subgroup difference in PICU length of stay was largely explained by the higher IMV use in the single-respiratory group. Since IMV is part of the Phoenix respiratory subscore, this attenuation likely reflects shared information rather than formal mediation.

**Conclusions:**

In septic children with the same Phoenix total score, different system involvement patterns yield substantially different outcomes and resource use. A respiratory subsystem score ≥2 signals high risk of prolonged PICU stay, largely explained by the greater need for invasive ventilation. The total Phoenix score alone is insufficient for resource planning; the respiratory component may provide additional information beyond the total score.

## 1 Introduction

Sepsis remains one of the most common and life-threatening conditions encountered in pediatric intensive care units (PICUs), defined as life-threatening organ dysfunction resulting from a dysregulated host response to infection (1). Global estimates from the 2017 Global Burden of Disease study placed the worldwide sepsis burden at approximately 48.9 million cases, of which nearly half—25.2 million—occurred in children and adolescents under 20 years of age (2). Mortality among children with severe sepsis has been reported to reach 25% (95% CI: 21.6%–28.8%) (3), and even in high-resource settings, case-fatality rates range from 16.4% to 22.7% (4). Early recognition, accurate risk stratification, and timely intervention therefore remain cornerstones of improving outcomes in this vulnerable population.

For years, the lack of a unified diagnostic framework hampered efforts to standardize pediatric sepsis care. The Sepsis-3 definitions, introduced in 2016, established the Sequential Organ Failure Assessment (SOFA) score of 2 or greater as the diagnostic threshold for sepsis in adults (1). However, because the SOFA score was originally derived from adult populations and does not incorporate age-specific adjustments, its direct application to children proved problematic. To address this gap, Matics and Sanchez-Pinto (5) validated a pediatric version of the SOFA score—the pSOFA—in a cohort of critically ill children, demonstrating its prognostic value in this population. Despite this advance, the pSOFA still carried certain limitations, including ambiguities in age-dependent variable definitions and practical difficulties in obtaining some laboratory parameters in younger patients (5). Meanwhile, the quick SOFA (qSOFA) tool, though useful in adult settings, consistently demonstrated poor sensitivity when applied to pediatric populations, limiting its utility as a screening instrument in this age group (6–8).

A major turning point came in 2024 with the publication of the Phoenix Sepsis Score, the first consensus-based diagnostic and risk-stratification tool specifically designed for children (9). The Phoenix score comprises four organ-system domains—respiratory, coagulation, circulatory, and neurological—quantified respectively by oxygenation indices (PaO₂/FiO₂ or SpO₂/FiO₂ ratio), platelet count and international normalized ratio (INR), vasoactive agent use along with serum lactate, and the Glasgow Coma Scale (GCS). A total score of 2 or higher establishes the diagnosis of sepsis. Validation in large, multinational cohorts has demonstrated that the Phoenix criteria outperform pSOFA in predicting mortality among children with suspected infection, with favorable performance across different age strata (9–11).

Nevertheless, the relative contribution of each Phoenix domain is likely to vary considerably across different PICU settings, a question that remains inadequately characterized. In infants and young children, septic shock more frequently presents with ventricular dysfunction and low cardiac output, making cardiovascular compromise especially prominent (12), whereas respiratory involvement is expected to dominate in those whose caseload is chiefly composed of severe pneumonia (13). Such population heterogeneity carries direct implications for how the Phoenix score should be interpreted and applied in resource allocation decisions. However, in a PICU setting where respiratory infections constitute the predominant admission diagnosis—a profile shared by many units worldwide—a critical question emerges: does an identical total Phoenix score reflect comparable resource needs and clinical trajectories when the underlying subsystem involvement profiles differ? Empirical evidence remains limited regarding Phoenix subsystem involvement patterns, their clinical correlates, and the predictive utility of individual domain scores for outcomes such as PICU length of stay.

In this single-center study, we therefore sought to characterize the distribution of subsystem involvement, compare clinical profiles across categories, and examine whether identical total scores could mask substantial differences in PICU stay depending on the systems affected, as well as the clinical factors associated with such differences, to inform more precise application of the Phoenix criteria in specific PICU contexts.

## 2 Methods

### 2.1 Study design and participants

This retrospective study was conducted at the PICU of Sichuan Provincial Maternity and Child Health Hospital, reviewing consecutive admissions from January 2024 to December 2025. Included were children aged 29 days to 18 years who met the Phoenix Sepsis Score criteria (total ≥ 2) within 24 h of admission. Exclusions comprised incomplete records precluding score calculation, death or discharge within 24 h, postoperative status, or primary hematologic disorders affecting coagulation. The final sample included 107 patients. The institutional ethics committee approved this chart review and waived informed consent (approval number: 20260812-315).

### 2.2 Phoenix sepsis score

The Phoenix Sepsis Score was calculated using the most abnormal values recorded within 24 h of PICU admission, following the 2024 criteria (9). The score encompasses four subsystems (respiratory, coagulation, circulatory, neurological).

Each subsystem was scored according to the Phoenix Sepsis Criteria (9). The respiratory subsystem (0–3 points) was assessed by the PaO₂/FiO₂ ratio (or SpO₂/FiO₂ when SpO₂ ≤ 97%) and invasive mechanical ventilation. The cardiovascular subsystem (0–6 points) comprised the sum of vasoactive-inotropic score, lactate level, and age-adjusted mean arterial pressure. The coagulation subsystem (0–2 points) was evaluated using platelet count, INR, D-dimer, and fibrinogen, with one point assigned for each abnormal parameter (maximum 2 points). The neurological subsystem (0–2 points) was assessed by Glasgow Coma Scale and pupillary response. The total Phoenix Sepsis Score was the sum of all subsystem scores, with a total ≥ 2 defining sepsis.

### 2.3 Group classification

Patients were stratified by the number of subsystems with a score ≥ 1. Because a total score ≥ 2 is required for sepsis diagnosis, the sole affected subsystem in single-system cases necessarily scored ≥ 2.

Single-system (*n* = 37): isolated respiratory (*n* = 29) or isolated coagulation (*n* = 8) involvement.

Dual-system (*n* = 62): exactly two subsystems— respiratory-coagulation (*n* = 59), coagulation-circulatory (*n* = 2), or respiratory-circulatory (*n* = 1).

Multi-system (*n* = 8): ≥ 3 subsystems involved.

For subgroup analyses, we compared the single-respiratory (*n* = 29) with the respiratory–coagulation (*n* = 59) subgroup—the most frequent patterns within each category. Both had a total Phoenix score of 2. The comparison between these two subgroups was *post hoc* and exploratory.

### 2.4 Data collection

Data extracted from electronic medical records included baseline demographics, comorbidities, Phoenix subsystem and total scores, age-adapted pSOFA score^3^, infection site, use and duration of invasive mechanical ventilation, routine laboratory parameters obtained within 24 h of admission, and clinical outcomes (PICU and total hospital stay, 28-day mortality).

Prolonged PICU stay was defined as ≥ 10 days, corresponding to the 75th percentile of the cohort distribution, a cut-off derived from the percentile-based approach commonly used in prior studies in the absence of a universally accepted standard for pediatric prolonged ICU stay (14, 15). In a sensitivity analysis, PICU length of stay was also analyzed as a continuous variable to avoid potential information loss from dichotomization.

### 2.5 Statistical analysis

Analyses were performed using R 4.3.2. Two-sided *P* < 0.05 was considered significant.

Continuous variables were summarized as medians (P25, P75) and compared using the Kruskal–Wallis H test (three groups) or Wilcoxon rank-sum test (two groups). Categorical variables were expressed as counts (percentages) and analyzed by chi-square or Fisher's exact test.

Multivariable logistic regression identified factors associated with prolonged PICU stay. The initial model included subgroup assignment, admission lactate, and age. These covariates were selected *a priori* based on clinical relevance. pSOFA was not included in the primary model because it is highly correlated with the Phoenix total score, and its inclusion would raise concerns about collinearity. A sensitivity model further added invasive mechanical ventilation (IMV) to examine whether the subgroup effect was attenuated after accounting for IMV use. Results are presented as odds ratios with 95% confidence intervals.

In a sensitivity analysis, PICU length of stay was analyzed as a continuous variable using log-linear regression, both without and with adjustment for IMV.

No *a priori* sample size calculation was performed; this was a retrospective analysis of all eligible consecutive cases. The subgroup analysis included 88 patients (29 single-respiratory, 59 respiratory–coagulation), with 30 events (prolonged PICU stay), meeting the recommended minimum of 5–10 events per variable for logistic regression (16).

## 3 Results

### 3.1 Patterns of system involvement and group classification

A total of 107 children with sepsis were enrolled and stratified according to the number of Phoenix Sepsis Score subsystems (respiratory, coagulation, circulatory, and neurological) with a score ≥ 1. Single-system involvement was identified in 37 patients (34.6%), among which isolated respiratory involvement was the most frequent (*n* = 29, 27.1%), followed by isolated coagulation involvement (*n* = 8, 7.5%). Dual-system involvement occurred in 62 patients (57.9%) and represented the predominant pattern; the respiratory-coagulation combination accounted for the vast majority of this category (*n* = 59, 55.1% of the total cohort, 95.2% of dual-system cases). The remaining dual-system combinations included coagulation-circulatory (*n* = 2, 1.9%) and respiratory-circulatory (*n* = 1, 0.9%). Multi-system involvement (≥ 3 subsystems) was observed in 8 patients (7.5%). Overall, respiratory and/or coagulation system involvement was present in 96 of 107 patients (89.7%), indicating that these two subsystems were the principal contributors to meeting the Phoenix Sepsis Score threshold in this cohort (Supplementary Table 1, Figure 1).

**Figure 1:**
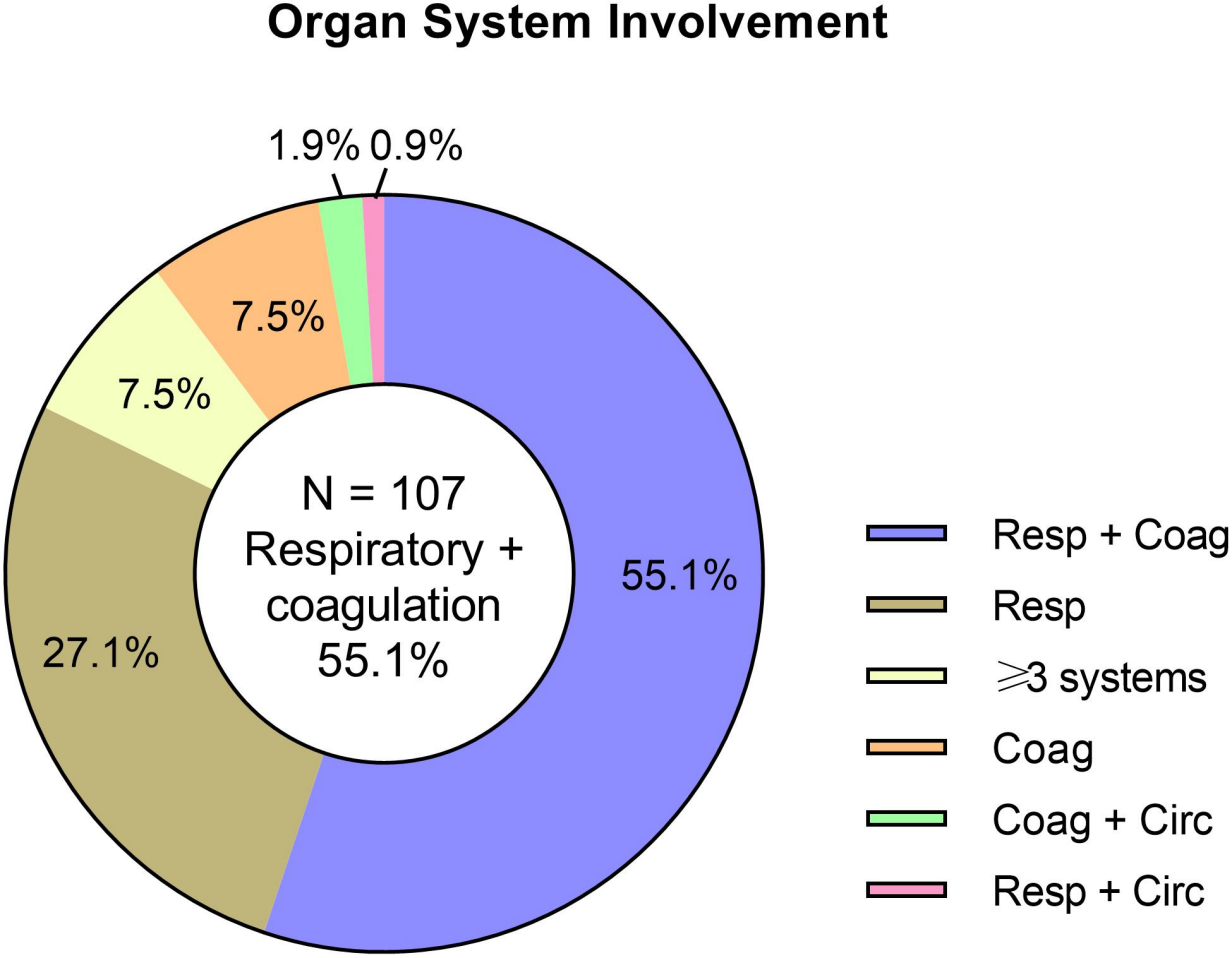
Distribution of system involvement patterns based on phoenix sepsis score subsystems in 107 children with sepsis.

### 3.2 Comparison of clinical characteristics across the three groups

Baseline demographics and comorbidities were comparable across the three groups (all *P* > 0.05). The single-system group had the highest rate and duration of invasive mechanical ventilation, whereas shock occurred exclusively in the multi-system and dual-system groups (*P* = 0.004). Multi-system involvement was associated with the most severe illness, as reflected by the highest total Phoenix and pSOFA scores, along with markedly elevated lactate, aminotransferases, urea, and WBC counts, and lower sodium levels (all *P* < 0.05). Respiratory tract infection was the predominant primary site in the single- and dual-system groups (> 80%), whereas multi-system cases showed greater diversity, including gastrointestinal and hematologic sources. Although the median total Phoenix scores were identical (2 points) between the single- and dual-system groups (*P* > 0.05), pSOFA scores differed significantly (*P* < 0.001), suggesting that the same Phoenix total may obscure broader organ dysfunction. Notably, the single-system group had the longest PICU and total hospital stays, the dual-system group intermediate, and the multi-system group the shortest (*P* = 0.002 and *P* = 0.001, respectively). Deaths were confined to the multi-system group (37.5%, *P* = 0.001) (Table 1).

**Table 1:** Clinical characteristics of the three groups stratified by number of affected phoenix subsystems.

Variable	Single-system (*n* = 37)	Dual-system (*n* = 62)	Multi-system (*n* = 8)	*P* value
Demographics				
Male sex, *n* (%)	27 (73.0)	39 (62.9)	4 (50.0)	0.394
Age, months	12.0 (3.0, 37.0)	12.0 (4.0, 46.0)	55.5 (9.5, 80.3)	0.272
Weight, kg	8.9 (5.7, 14.0)	9.4 (5.8, 15.4)	17.5 (9.1, 24.8)	0.273
Comorbidities				
Congenital heart disease, *n* (%)	1 (2.7)	7 (11.3)	0 (0)	0.347
History of prematurity, *n* (%)	6 (16.2)	13 (21.0)	1 (12.5)	0.862
Organ involvement & treatment				
Invasive mechanical ventilation, *n* (%)	31 (83.8)	17 (27.4)	5 (62.5)	< 0.001
Shock, *n* (%)	0 (0)	4 (6.5)	3 (37.5)	0.004
Duration of invasive ventilation, days	5.0 (1.0, 6.0)	0.0 (0.0, 0.8)	2.5 (0.0, 3.3)	< 0.001
Disease severity				
Total Phoenix Sepsis Score	2.0 (2.0, 2.0)	2.0 (2.0, 2.0)	4.5 (3.0, 7.5)	< 0.001
pSOFA score	3 (3, 4)	2 (1, 3)	5 (2.75, 11.25)	< 0.001
Primary infection site, n (%)				0.002
– Respiratory	30 (81.1)	54 (87.1)	3 (37.5)	
– Gastrointestinal	5 (13.5)	3 (4.8)	2 (25.0)	
Inflammatory markers				
WBC,×10⁹/L	7.3 (5.1, 11.7)	10.5 (8.3, 13.7)	19.0 (11.7, 22.4)	0.001
CRP, mg/L	8.9 (3.2, 40.2)	8.3 (3.4, 35.8)	31.6 (14.8, 54.5)	0.389
PCT, ng/mL	0.29 (0.11, 1.09)	0.29 (0.10, 1.91)	1.04 (0.51, 2.25)	0.405
Organ function parameters				
ALT, U/L	22.0 (17.0, 37.0)	21.0 (15.0, 39.0)	172.5 (24.8, 584.8)	0.033
AST, U/L	33.0 (28.0, 57.0)	40.0 (33.5, 57.5)	133.0 (53.5, 612.8)	0.010
Urea, mmol/L	2.6 (2.3, 4.1)	3.5 (2.9, 5.1)	4.4 (3.8, 11.5)	0.011
Lactate, mmol/L	1.21 (1.05, 1.76)	1.65 (1.28, 2.54)	5.30 (4.20, 5.71)	< 0.001
Sodium, mmol/L	136.0 (135.2, 138.6)	136.4 (135.2, 139.0)	133.9 (129.3, 135.6)	0.033
Clinical outcomes				
Total hospital stay, days	13.0 (11.0, 15.0)	10.0 (8.0, 12.0)	8.5 (3.8, 10.5)	0.001
PICU length of stay, days	10.0 (6.0, 12.0)	6.0 (4.0, 8.0)	4.5 (3.0, 5.5)	0.002
Mortality, *n* (%)	0 (0)	0 (0)	3 (37.5)	0.001

Continuous variables are presented as median (P25, P75); categorical variables as *n* (%). Comparisons among the three groups were performed using the Kruskal–Wallis H test for continuous variables and the chi-square test or Fisher's exact test for categorical variables. *P* < 0.05 was considered statistically significant.

WBC, white blood cell count; CRP, C-reactive protein; PCT, procalcitonin; ALT, alanine aminotransferase; AST, aspartate aminotransferase; PICU, pediatric intensive care unit; pSOFA, pediatric Sequential Organ Failure Assessment.

### 3.3 Phoenix subsystem score composition across the three groups

Detailed Phoenix subsystem scores and total scores for each group are presented in Table 2 and visualized in Figure 2. Across all four subsystem scores and the total score, differences among the three groups reached statistical significance (Kruskal–Wallis test, all *P* < 0.001).

**Table 2:** Phoenix subsystem score components across the three groups.

Subsystem	Single-system (*n* = 37)	Dual-system (*n* = 62)	Multi-system (*n* = 8)	*P* value*
Respiratory	2 (2, 2)	1 (1, 1)	1 (1, 2)	< 0.001
Coagulation	0 (0, 0)	1 (1, 1)	1.5 (1, 2)	< 0.001
Circulatory	0 (0, 0)	0 (0, 0)	1 (1, 2.75)	< 0.001
Neurological	0 (0, 0)	0 (0, 0)	0.5 (0, 1)	< 0.001
Total Phoenix Sepsis Score	2 (2, 2)	2 (2, 2)	4.5 (3, 7.5)	< 0.001

Data are presented as median (P25, P75).

*P values were derived from the Kruskal–Wallis H test across the three groups.

**Figure 2:**
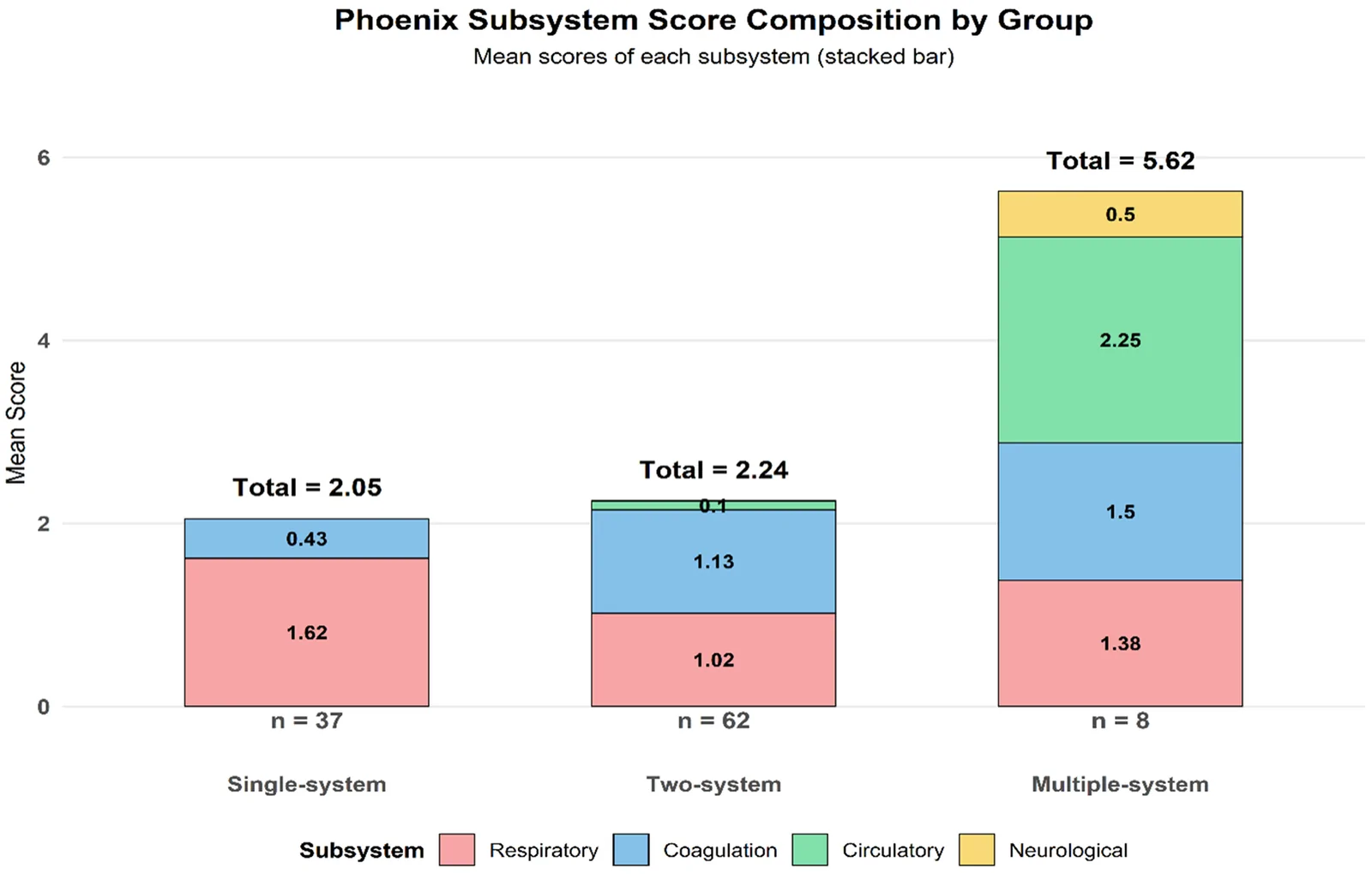
Phoenix subsystem score composition by group.

As shown in Figure 2, the total score in the single-system group derived almost entirely from the respiratory subsystem (median 2, mean 1.62), with only a minor contribution from coagulation (mean 0.43). In the dual-system group, the total score was jointly driven by respiratory and coagulation subsystems (median 1 for both; means 1.02 and 1.13, respectively), which contributed to a comparable extent. The multi-system group exhibited the highest total score (median 4.5), with involvement across all four subsystems; among these, the circulatory subsystem showed the most pronounced elevation (mean 2.25), followed by coagulation (1.50), respiratory (1.38), and neurological (0.50) subsystems, underscoring the greater illness severity in this group. Overall, both subsystem-specific and total scores increased progressively with the number of affected systems.

### 3.4 Subgroup analysis: single-respiratory versus respiratory–coagulation involvement

Given that the single-system and dual-system groups had identical median total Phoenix scores (2 points) but significantly different PICU lengths of stay (*P* < 0.001), we conducted a focused subgroup analysis comparing the most frequent patterns within each category: the single-respiratory subgroup (*n* = 29) and the respiratory–coagulation subgroup (*n* = 59) (Table 3). Although the total Phoenix score was 2 (2, 2) in both subgroups (*P* = 0.457), pSOFA scores were significantly higher in the single-respiratory subgroup (*P* < 0.001). Invasive mechanical ventilation was required in all single-respiratory patients, with a median duration of 6 days, versus 25.4% and 0 days in the respiratory–coagulation subgroup (both *P* < 0.001). Laboratory findings showed that WBC, urea, and lactate levels were significantly lower in the single-respiratory subgroup (all *P* < 0.05), whereas CRP, PCT, ALT, AST, and sodium did not differ significantly between the two groups. Both total hospital stay and PICU stay were significantly longer in the single-respiratory subgroup (both *P* < 0.001), with no mortality observed in either group. No significant between-group differences were found in shock prevalence, primary infection site, or other variables listed in Table 3.

**Table 3:** Comparison of clinical characteristics between the single-respiratory and respiratory-coagulation subgroups.

Variable	Single-respiratory (*n* = 29)	respiratory-coagulation (*n* = 59)	*P* value
Demographics			
Male sex, *n* (%)	21 (72.4)	37 (62.7)	0.507
Age, months	5 (3, 27)	12 (4, 39)	0.216
Weight, kg	7.3 (5.5, 14.5)	9.3 (5.85, 14.8)	0.613
Organ system involvement and treatment			
Invasive mechanical ventilation, *n* (%)	29 (100)	15 (25.4)	< 0.001
Duration of invasive ventilation, days	6 (4, 7)	0 (0, 0)	< 0.001
Disease severity			
Total Phoenix Sepsis Score	2 (2, 2)	2 (2, 2)	0.457
pSOFA score	3 (3, 3)	2 (1, 3)	< 0.001
Inflammatory markers			
WBC,×10⁹/L	7.27 (5.12, 11.72)	10.47 (8.27, 13.61)	0.023
Organ function parameters			
Urea, mmol/L	2.6 (2.2, 3.7)	3.5 (2.9, 4.5)	0.041
Lactate, mmol/L	1.25 (1.08, 1.46)	1.58 (1.27, 2.41)	0.008
Length of stay and outcomes			
Total hospital stay, days	14 (11, 15)	10 (8, 12)	< 0.001
PICU length of stay, days	10 (9, 12)	6 (4, 8)	< 0.001

Data are presented as median (P25, P75) for continuous variables and as *n* (%) for categorical variables. Group comparisons were performed using the Wilcoxon rank-sum test, chi-square test, or Fisher's exact test as appropriate. Both subgroups had identical median total Phoenix Sepsis Scores of 2 (2, 2) (*P* = 0.457). No significant between-group differences were observed for congenital heart disease, history of prematurity, shock, primary infection site, CRP, PCT, ALT, AST, or sodium (all *P* > 0.05); these variables are not shown.

ALT, alanine aminotransferase; AST, aspartate aminotransferase; CRP, C-reactive protein; PCT, procalcitonin; PICU, pediatric intensive care unit; pSOFA, pediatric Sequential Organ Failure Assessment; WBC, white blood cell count.

### 3.5 Multivariable logistic regression for prolonged PICU stay

Prolonged PICU stay (≥ 10 days, the 75th percentile of the cohort) occurred in 65.5% (19/29) of the single-respiratory subgroup versus 18.6% (11/59) of the respiratory–coagulation subgroup.

Multivariable logistic regression, adjusted for admission lactate and age, identified the single-respiratory subgroup as a significant predictor of prolonged stay (OR = 6.92, 95% CI: 2.48–20.80, *P* < 0.001), whereas lactate and age were not (Table 4). After further adjustment for invasive mechanical ventilation, IMV emerged as the strongest risk factor (OR = 18.80, 95% CI: 3.78–123.24, *P* < 0.001), and age became marginally significant (OR = 0.98, 95% CI: 0.95–0.996, *P* = 0.037). The subgroup effect was attenuated from OR = 6.92 to 1.27 (*P* = 0.739), which is consistent with the clinical pathway in which the greater need for IMV in the single-respiratory subgroup is associated with their longer PICU stay (Table 4, Figure 3).

**Table 4:** Multivariable logistic regression analysis of prolonged PICU stay (≥ 10 days).

Variable	Multivariable model^a^ OR (95% CI)	*P* value	Sensitivity model^b^ OR (95% CI)	*P* value
Group (reference: respiratory-coagulation)				
– Single-respiratory	6.92 (2.48–20.80)	< 0.001	1.27 (0.30–5.07)	0.739
Admission lactate, mmol/L	0.58 (0.27–1.10)	0.121	0.79 (0.34–1.74)	0.570
Age, months	0.98 (0.95–1.00)	0.076	0.98 (0.95–0.996)	0.037
Invasive mechanical ventilation (yes vs. no)	NA	NA	18.80 (3.78–123.24)	< 0.001

CI, confidence interval; PICU, pediatric intensive care unit; OR, odds ratio.

a The multivariable model was adjusted for admission lactate and age.

b The sensitivity model further adjusted for invasive mechanical ventilation. This attenuation should be interpreted as exploratory and non-causal, because IMV is embedded in the Phoenix respiratory subscore.

**Figure 3:**
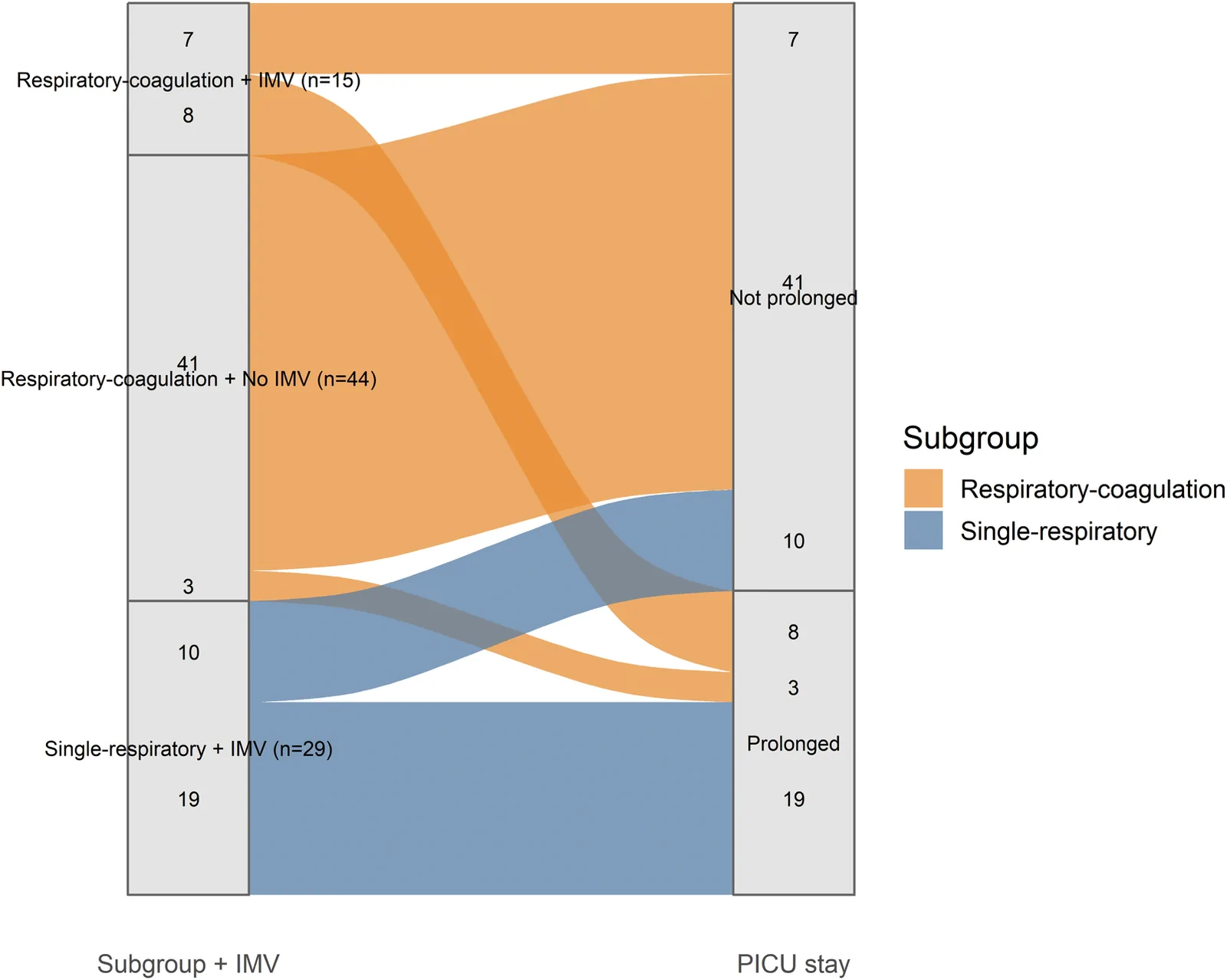
Exploratory diagram showing the distribution of invasive mechanical ventilation (IMV) use and prolonged pediatric intensive care unit (PICU) stay across subgroups.

In a sensitivity analysis with PICU length of stay as a continuous variable, the subgroup effect was significant before adjustment for IMV (exp(β) = 1.60, 95% CI: 1.25–2.04, *P* < 0.001) but not after adjustment (exp(β) = 0.917, 95% CI: 0.690–1.21, *P* = 0.533). IMV use remained significantly associated with longer PICU stay (exp(β) = 2.20, 95% CI: 1.68–2.88, *P* < 0.001) (Supplementary Table 2).

The pie chart illustrates the composition of patients grouped by the number and combination of affected subsystems (respiratory, coagulation, circulatory, and neurological) according to the Phoenix Sepsis Score. Single-system involvement included respiratory alone (Resp, 27.1%, *n* = 29) and coagulation alone (Coag, 7.5%, *n* = 8). Among dual-system involvement, the respiratory-coagulation combination was the most prevalent (Resp + Coag, 55.1%, *n* = 59), followed by coagulation-circulatory (Coag + Circ, 1.9%, *n* = 2) and respiratory-circulatory (Resp + Circ, 0.9%, *n* = 1). Multi-system involvement (≥3 systems) accounted for 7.5% (*n* = 8). Overall, respiratory and/or coagulation system involvement accounted for 89.7% (96/107) of all cases, indicating that these two systems were the predominant contributors to meeting the Phoenix Sepsis Score criteria in this cohort.

Stacked bar chart showing mean scores of each subsystem across the three groups. The single-system group (*n* = 37) was dominated by respiratory system involvement (mean 1.62), with a small contribution from the coagulation system (mean 0.43). The dual-system group (*n* = 62) exhibited involvement of both respiratory (mean 1.02) and coagulation (mean 1.13) systems, with minimal circulatory involvement (mean 0.10). The multiple-system group (*n* = 8) showed involvement across all four subsystems, with the highest circulatory score (mean 2.25), followed by coagulation (mean 1.50), respiratory (mean 1.38), and neurological (mean 0.50) systems. The total bar height represents the mean total Phoenix Sepsis Score for each group (2.05, 2.24, and 5.62, respectively). Numbers within bars indicate the mean score of each subsystem.

The single-respiratory subgroup (*n* = 29) universally required IMV; 19 of 29 (65.5%) had prolonged PICU stay (≥10 days), and 10 of 29 (34.5%) did not. In the respiratory-coagulation subgroup (*n* = 59), 15 (25.4%) required IMV, of whom 8 (53.3%) had prolonged stay and 7 (46.7%) did not; among the 44 who did not require IMV, 3 (6.8%) had prolonged stay and 41 (93.2%) did not. This pathway should be interpreted as exploratory.

## 4 Discussion

This study analyzed the patterns of subsystem involvement and clinical characteristics in 107 children with sepsis admitted to the PICU, using the Phoenix Sepsis Score as the classification framework. Three principal findings emerged. First, the respiratory and coagulation systems were the primary contributors to Phoenix score positivity. Second, the respiratory–coagulation dual-system pattern was the most frequently observed combination. Third, among children with identical total Phoenix scores, different subsystem involvement profiles were associated with markedly different PICU length of stay and resource utilization, largely explained by the need for invasive mechanical ventilation.

### 4.1 Predominance of respiratory and coagulation system involvement

Respiratory system involvement, either in isolation or in combination with other systems, was present in 83.2% of the cohort and represented the single largest contributor to Phoenix score attainment. Respiratory tract infection constitutes the primary source of sepsis in approximately 40%–50% of affected children (17), a figure consistent with the epidemiology of pediatric sepsis. The respiratory domain of the Phoenix score uses the PaO₂/FiO₂ or SpO₂/FiO₂ ratio, which offers greater objectivity and cross-setting applicability than earlier criteria (9–11). In our single-respiratory subgroup, the median respiratory score was 2, corresponding to moderate-to-severe hypoxemia—a finding that aligned with the 100% rate of invasive mechanical ventilation observed in this group.

Coagulation abnormalities were equally prevalent in this cohort. Isolated coagulation involvement was sufficient to meet the Phoenix diagnostic threshold in 8 cases (7.5%), underscoring the diagnostic relevance of coagulation derangements in pediatric sepsis. Further analysis revealed that 65.4% of patients had involvement of two or more subsystems, with the respiratory–coagulation combination accounting for 55.1% of all cases—the most frequent pattern overall. Several factors may explain this predominance. The interplay between inflammation and coagulation is well recognized: inflammatory cytokines activate the coagulation cascade, while thrombin reciprocally amplifies pro-inflammatory signals, establishing a self-perpetuating immunothrombotic loop (18, 19). In addition, the Phoenix coagulation domain has a relatively low scoring threshold (INR > 1.3 or platelet count < 100 × 10^3^/*μ*L yields 1 point), enabling early identification of children with even mild coagulation abnormalities in the setting of pre-existing respiratory involvement. This may confer higher diagnostic sensitivity compared with the earlier IPSCC 2005 criteria (20). Collectively, these observations suggest that by treating respiratory and coagulation dysfunction as parallel and independent dimensions, the Phoenix score effectively captures the dominant sepsis phenotype in PICU settings where respiratory infection is the predominant admission diagnosis, a finding consistent with its reported high diagnostic yield in children with suspected infection (21, 22).

### 4.2 Multi-system involvement and prognosis

The mortality rate in the multi-system group (37.5%) was significantly higher than in the single-system and dual-system groups (0% in both), with a median total Phoenix score of 4.5 and marked elevations in lactate and liver enzymes, consistent with the well-established association between multi-organ dysfunction and adverse outcomes in sepsis (3). In the original Phoenix validation study, which included over 3,000 children with suspected infection, total scores ≥ 2 were associated with significantly higher in-hospital mortality, and each 1-point increment conferred an incremental increase in death risk (10). A separate validation study conducted in a pediatric emergency department setting further demonstrated that the Phoenix score outperformed the IPSCC criteria in predicting 3-day, 28-day, and overall in-hospital mortality (23). Together, these findings indicate that the total Phoenix score effectively discriminates illness severity across the sepsis spectrum.

However, a notable observation emerged from the comparison between the single-system and dual-system groups. Although both groups had identical median total Phoenix scores of 2 points (*P* > 0.05), the single-system group had significantly higher pSOFA scores [3 (3, 4) vs. 2 (1, 3), *P* < 0.001] and prolonged PICU stay [10.0 (6.0, 12.0) vs. 6.0 (4.0, 8.0) days, *P* = 0.002]. This discrepancy suggests that identical Phoenix total scores may obscure meaningful differences in underlying organ dysfunction and clinical trajectory. Of note, circulatory and neurological involvement were uncommon in this cohort, with only 3 cases (2.8%) in the dual-system group involving the circulatory subsystem. This is likely a reflection of the center's predominant admission diagnosis of severe pneumonia, where patients were captured by the Phoenix score at an early disease stage, before overt circulatory failure or neurological impairment had developed. Under the Phoenix framework, circulatory scores rise only with higher illness severity, given that they incorporate lactate levels and vasoactive requirements as key indicators (9, 11).

### 4.3 Clinical factors associated with divergent outcomes despite identical total phoenix scores

To further explore the factors related to the outcome divergence described above, we compared the two most representative subgroups—those with isolated respiratory involvement (*n* = 29) and those with combined respiratory–coagulation involvement (*n* = 59). Although total Phoenix scores were 2 (2, 2) in both subgroups (*P* = 0.457), marked differences emerged in PICU length of stay [10 (9, 12) vs. 6 (4, 8) days, *P* < 0.001] and rates of invasive mechanical ventilation (100% vs. 25.4%, *P* < 0.001).

A respiratory score ≥ 2, as observed in the single-respiratory subgroup, reflects moderate-to-severe hypoxemia or respiratory failure warranting ventilatory support. The initiation of invasive ventilation entails prolonged sedation, frequent ventilator adjustments, weaning protocols, and close monitoring for complications—all of which may contribute to PICU stay (14, 17). This group had a 100% ventilation rate and a median ventilation duration of 6 days. By contrast, the respiratory–coagulation group had less severe respiratory involvement (score 1), with only 25.4% requiring ventilation and a median duration of 0 days, which may explain the substantially shorter stay. It is noteworthy that pSOFA scores were significantly higher in the single-respiratory subgroup [3 (3, 3) vs. 2 (1, 3), *P* < 0.001], confirming greater illness severity. However, pSOFA serves as a marker of severity rather than a direct consumer of PICU resources; the factor most closely related to extended stay is the invasive ventilation required to support failing respiratory function. Invasive ventilation was the factor most strongly linking illness severity (as reflected by pSOFA) to prolonged PICU stay.

Multivariable logistic regression identified IMV as the factor most strongly associated with prolonged PICU stay (≥10 days) (OR = 18.80, 95% CI: 3.78–123.24, *P* < 0.001). When IMV was added to the model, the subgroup effect was attenuated from OR = 6.92 (95% CI: 2.48–20.80, *P* < 0.001) to OR = 1.27 (95% CI: 0.30–5.07, *P* = 0.739). A sensitivity analysis using continuous PICU length of stay showed a consistent attenuation of the subgroup effect. Because IMV is part of the Phoenix respiratory subscore, this attenuation most likely reflects shared information between the subgroup variable and IMV rather than a formally estimated mediating effect.

These findings are consistent with a body of prior evidence. Marcin et al. (14) were among the first to define prolonged PICU stay as > 12 days and identified mechanical ventilation as one of its strongest predictors. Moya-Sánchez et al. (24) quantified this relationship, reporting a 100% ventilation rate among children with PICU stay > 14 days (vs. 77.2% in the non-prolonged group, *P* < 0.001), with ventilation conferring a 2.37-fold increased risk (OR = 2.37) of prolonged stay. Yang et al. (25), from the perspective of chronic critical illness, identified ventilation duration as an independent risk factor for progression to chronic critical illness (defined as PICU stay ≥ 14 days) in children with sepsis. In a multicenter cohort of 9,675 children with severe sepsis, Watson et al. (17) reported a mean length of stay of 31.7 days among those requiring invasive mechanical ventilation, compared with 16.6 days for those without—a difference accompanied by substantially higher resource consumption. Odetola et al. (26), using a national inpatient database, further demonstrated that illness severity and multi-organ dysfunction were key determinants of prolonged stay and increased resource utilization. Across different countries, study designs, and time periods, these converging lines of evidence point to a consistent conclusion: invasive mechanical ventilation is closely and robustly associated with extended PICU stay.

These observations carry important clinical implications. Children with identical total Phoenix scores may differ considerably in resource consumption and length of stay, and the respiratory subsystem score may provide additional information beyond the total score for PICU resource utilization. Identifying septic children with a respiratory score ≥ 2 may enable earlier recognition of high-resource needs and facilitate more efficient allocation of PICU beds and staffing. Future studies should investigate whether integrating standardized measures of nursing complexity and intensity with medical severity scores can capture additional dimensions of care needs and resource utilization, thereby improving the characterization of length of stay and resource requirements across patient profiles (15, 27–30). In addition, the observed association between younger age and prolonged PICU stay (OR = 0.98, 95% CI: 0.95–0.996, *P* = 0.037) deserves attention, as it may reflect age-specific anatomical and physiological features of the respiratory system and greater difficulty with weaning in infants (31).

### 4.4 Limitations

Several limitations warrant consideration. First, this was a single-center retrospective study with a relatively modest sample size, particularly for the multi-system group (*n* = 8). In addition, PICU length of stay is influenced by non-clinical factors such as discharge policies, bed availability, and local practice patterns. These factors may limit the generalizability of our findings to settings with different healthcare resources. Second, the Phoenix score was assessed only at a single time point (within 24 h of admission), and we did not capture serial changes in scores over the course of illness. Third, the definition of prolonged PICU stay (≥ 10 days) was based on the 75th percentile of our cohort distribution. Although this approach follows the methodological framework used in prior study (14), variations in cutoff values across studies may affect comparability. Fourth, the extended Phoenix-8 criteria—which include renal and hepatic dimensions—were not incorporated in this analysis, and residual confounding by unmeasured severity, including pSOFA, cannot be fully excluded. Fifth, because IMV is embedded in the Phoenix respiratory subscore, the observed association should be interpreted as descriptive rather than causal. Multicenter prospective studies, or studies with longer enrollment periods at our center, are needed to confirm and extend our findings.

## 5 Conclusions

In this cohort of children with sepsis, the respiratory and coagulation systems were the predominant contributors to a positive Phoenix Sepsis Score. However, identical total Phoenix scores did not translate into comparable clinical outcomes or resource utilization. A respiratory subsystem score ≥ 2—reflecting moderate-to-severe respiratory failure warranting invasive mechanical ventilation—emerged as a key factor associated with prolonged PICU stay. Invasive ventilation was the clinical factor most strongly linked to extended length of stay. These findings suggest that the total Phoenix score alone is insufficient for estimating resource needs; the respiratory subsystem score provides additional information beyond the aggregate score. Identifying children with a respiratory score ≥ 2 may facilitate earlier recognition of high-resource demand and enable more efficient allocation of PICU beds and staffing.

## Data Availability

The raw data supporting the conclusions of this article will be made available by the authors, without undue reservation.
