# Supplementary material for: Divergent PICU lengths of stay despite identical phoenix sepsis scores: heterogeneity in pediatric sepsis outcomes according to system involvement profiles
Source: Front Pediatr. 2026 Oct 21;14:1986840. doi: 10.3389/fped.2026.1986840 (PMC13731310; doi:10.3389/fped.2026.1986840)
Supplement: Supplementary file 1 [file Table1.docx]

**Supplementary Table 1. Distribution of System Involvement Patterns Based on Phoenix Sepsis Score Subsystems**

| Involvement pattern | n (%) | Notes |
| --- | --- | --- |
| Single-system involvement | 37 (34.6) |  |
| – Respiratory alone | 29 (27.1) | Respiratory subsystem score ≥ 2, all other subsystems = 0 |
| – Coagulation alone | 8 (7.5) | Coagulation subsystem score ≥ 2, all other subsystems = 0 |
| Dual-system involvement | 62 (57.9) |  |
| – Respiratory + coagulation | 59 (55.1) | Most frequent dual-system combination |
| – Coagulation + circulatory | 2 (1.9) |  |
| – Respiratory + circulatory | 1 (0.9) |  |
| Multi-system involvement (≥ 3 systems) | 8 (7.5) | Involvement of ≥ 3 subsystems with score ≥ 1 |

Note: Each Phoenix subsystem score ranges from 0 to 3 points; "involvement" was defined as a subsystem score ≥ 1. Because a total Phoenix score ≥ 2 is required for sepsis diagnosis, the sole affected subsystem in the single-system group necessarily had a score ≥ 2.
