# Supplementary material for: Divergent PICU lengths of stay despite identical phoenix sepsis scores: heterogeneity in pediatric sepsis outcomes according to system involvement profiles
Source: Front Pediatr. 2026 Oct 21;14:1986840. doi: 10.3389/fped.2026.1986840 (PMC13731310; doi:10.3389/fped.2026.1986840)
Supplement: Supplementary file 2 [file Table2.docx]

**Supplementary Table 2. Log-linear regression analysis of continuous PICU length of stay**

| Variable | Model 1 (without IMV) exp(β) (95% CI) | P value | Model 2 (with IMV) exp(β) (95% CI) | P value |
| --- | --- | --- | --- | --- |
| Group (reference: respiratory-coagulation) |  |  |  |  |
| – Single-respiratory | 1.60 (1.25–2.04) | < 0.001 | 0.917 (0.690–1.21) | 0.533 |
| Admission lactate, mmol/L | 0.933 (0.820–1.06) | 0.285 | 1.01 (0.905–1.14) | 0.815 |
| Age, months | 0.996 (0.993–0.999) | 0.012 | 0.995 (0.993–0.998) | < 0.001 |
| IMV (yes vs. no) | NA | NA | 2.20 (1.68–2.88) | < 0.001 |

The dependent variable was log-transformed PICU length of stay. Results are presented as exp(β) with 95% confidence intervals, interpreted as geometric multiples. Model 1 was adjusted for group, lactate, and age. Model 2 was further adjusted for invasive mechanical ventilation.

Abbreviations: CI, confidence interval; IMV, invasive mechanical ventilation; NA, not applicable; PICU, pediatric intensive care unit.
